# Acid Ceramidase Protects Against Hepatic Ischemia/Reperfusion Injury by Modulating Sphingolipid Metabolism and Reducing Inflammation and Oxidative Stress

**DOI:** 10.3389/fcell.2021.633657

**Published:** 2021-05-06

**Authors:** Yuan Jiang, Xingxuan He, Calogera M. Simonaro, Bin Yi, Edward H. Schuchman

**Affiliations:** ^1^Department of Clinical Laboratory, Xiangya Hospital, Central South University, Changsha, China; ^2^Department of Clinical Laboratory, Hunan Cancer Hospital and The Affiliated Cancer Hospital of Xiangya School of Medicine, Central South University, Changsha, China; ^3^Department of Genetics and Genomic Sciences, Icahn School of Medicine at Mount Sinai, New York, NY, United States

**Keywords:** ischemia reperfusion, liver injury, acid ceramidase, ceramide, sphingolipids

## Abstract

Ceramide is a bioactive signaling lipid involved in the pathogenesis of numerous diseases. It also plays an important role in ischemia reperfusion (IR) injury via activation of inflammatory/oxidative stress-stimulated signaling pathways, resulting in tissue damage. Acid ceramidase is a lipid hydrolase that modulates the levels of ceramide, and as such has a potential therapeutic role in many human diseases where ceramide has been implicated. Here we investigated the therapeutic potential of recombinant acid ceramidase in a murine model of hepatic IR injury. Serum ALT, AST, and LDH activities, as well as oxidative stress (MDA) and inflammatory (MCP-1) markers, were increased in mice subjected to IR compared to a sham group. In contrast, these elevations were significantly lower in an IR group pretreated with a single injection of acid ceramidase. Histological examination by two different assessment criteria also revealed that acid ceramidase pretreatment alleviated IR-induced hepatocyte damage, including reduced evidence of cell death and necrosis. In addition, elevated ceramide and sphingosine levels were observed in the IR group compared to sham, and were markedly reduced when pretreated with acid ceramidase. In contrast, the levels of the protective signaling lipid, sphingosine-1-phosphate (S1P), were reduced following IR and elevated in response to acid ceramidase pretreatment. These changes in sphingolipid levels could be correlated with changes in the activities of several sphingolipid-metabolizing enzymes. Overall, these results indicated that sphingolipid changes were an important pathologic component of hepatic IR injury, and that acid ceramidase administration ameliorated these lipid changes and other downstream pathologic changes.

## Introduction

Hepatic ischemia reperfusion (IR) injury is a common pathological process that occurs during various surgical procedures, including liver resection and transplantation, as well as in response to trauma, hemorrhagic shock, cancer and other clinical insults. It is characterized by first the interruption, and then re-establishment, of the liver blood flow and oxygen supply (Selzner et al., 2003; Karatzas et al., 2014; He and Schuchman, 2018). Restoration of blood supply should protect the liver from injury; however, paradoxically such reperfusion following ischemia often leads to further hepatocyte death and tissue damage.

A large number of studies have revealed the complex mechanisms underlying hepatic IR injury. Briefly, during ischemia aerobic metabolism is interrupted due to the lack of oxygen supply. Build-up of oxidative cell damage further occurs during reperfusion to the ischemic area, which is characterized by excess reactive oxygen species (ROS) release and inflammatory cytokine recruitment (Chan, 2001; Unal et al., 2017). It is well known that mitochondria are the primary ROS generation sites under normal physiologic conditions. In the case of IR, mitochondrial dysfunction appears to result in suppression of the respiratory chain and elevation of ROS formation (Won and Singh, 2006). Meanwhile, activated Kupffer and dendritic cells release pro-inflammatory cytokines such as monocyte chemoattractant protein 1 (MCP-1) and tumor necrosis factor alpha (TNFα) (Perry et al., 2011; Shuh et al., 2013). Even though it is widely accepted that IR injury involves the production of ROS, recruiting inflammatory mediators to the injured tissue, other downstream mechanisms contribute to the damage and remain to be fully elucidated.

Over two decades of research has documented that the sphingolipid, ceramide, is a key bioactive signaling molecule that mediates the proliferation, survival and death of cells. Ceramide accumulates in response to diverse cellular stresses (e.g., inflammation, oxidative stress, apoptosis, infection, and others) (Gulbins and Kolesnick, 2002; Zeidan and Hannun, 2010; He and Schuchman, 2012), and contributes to the pathology of many diseases (e.g., diabetes, cardiovascular disease, and Alzheimer’s disease) (He et al., 2010; Chavez and Summers, 2012; Pan et al., 2014; He and Schuchman, 2018). The generation of ceramide is from two major pathways, sphingomyelin hydrolysis and *de novo* biosynthesis. For example, diverse stress signals induce the rapid expression of sphingomyelinases (SMases), resulting in sphingomyelin hydrolysis and ceramide formation (Haimovitz-Friedman et al., 1997). Other studies have shown that ceramide generation by SMases contributes to ROS and TNFα induced cell death and tissue damage (Suematsu et al., 2003; Won and Singh, 2006).

The specific role of ceramide in the pathogenesis of IR injury has attracted considerable attention since the first report by Bradham et al. (1997), and a growing body of evidence has shown that the accumulation of ceramide occurs in multiple models of ischemia and reperfusion injury (Kubota et al., 1996; Nakane et al., 2000; Ramirez-Camacho et al., 2016). For example, the release of TNFα and activation of SMases was observed during the reperfusion of the ischemic liver (Alessenko et al., 2002). Ceramide and TNFα are known to induce ROS generation, which in turn amplifies ROS/TNFα-ceramide cycling and exacerbates IR injury (Suematsu et al., 2003; Lecour et al., 2006). Notably, inhibition of SMases decreased ceramide formation during hepatic IR and mitigated cell necrosis (Tuzcu et al., 2017), while administration of a ceramidase inhibitor enhanced ceramide generation and exacerbated hepatic IR injury (Llacuna et al., 2006).

These findings suggest that ceramide generated from TNFα/ROS mediated activation of SMases is central to the pathogenesis of IR-induced liver injury, and that the modulation of ceramide levels may be a therapeutic strategy to prevent or treat IR injury in diverse clinical settings. In this paper we investigated the effect of a specific ceramide hydrolase, acid ceramidase (AC, E.C. #3.5.1.23), on liver IR injury in mice, and assessed its therapeutic value in protecting the liver against ceramide toxicity due to IR.

## Materials and Methods

### Reagents

The aspartate aminotransferase (AST) microplate test kit was purchased from BioAssay Systems LLC (Hayward, CA, United States), the alanine aminotransferase (ALT) microplate test kit was from Thermo Scientific Inc (Waltham, MA, United States), and the lactate dehydrogenase (LDH) microplate test kit was from BioVision Inc. (Milpitas, CA, United States). The malondialdehyde (MDA) assay kit and sphingosine standard were from Cayman Chemical (Ann Arbor, MI, United States). The mouse MCP-1 quantikine ELISA kit was from R&D Systems Inc. (Minneapolis, MN, United States). Recombinant human AC was purified from an overexpressed Chinese hamster ovary (CHO) cell line using an A plus (AKTA) system as previously described (He et al., 2003b). This recombinant enzyme effectively degrades ceramide within cells, and is also efficiently delivered to the liver after systemic administration to mice, leading to ceramide degradation (He et al., 2017). Optimal uptake of the enzyme into the liver was found at 12–24 h post-administration. The NBD-sphingosine and sphingosine-1-phosphate (S1P) were from Avanti Polar Lipids, Inc. (Alabaster, AL, United States). The Bodipy-FL C12 sphingomyelin and naphthalene-2,3-dicarboxyaldehyde (NDA) were from Life Technologies (Carlsbad, CA, United States).

### Animals

Wild-type C57BL/6J mice (12 weeks) were purchased from Charles River (Wilmington, MN, United States). All experiments in this study were performed at the Icahn School of Medicine under a protocol (#98-0089) approved by the Institutional Animal Care and Use Committee, and adhered to the NIH guidelines for the use of experimental animals. All mice were fed *ad libitum* food and water, and kept under constant environmental conditions with a 12-h light-dark cycle.

Mice were randomly divided into four groups (*n* = 10 per group): (1) sham group: mice were subjected to surgery without vascular occlusion; (2) IR group: the mice were subjected to surgery with vascular occlusion; (3) IR + AC5 group: mice were injected intraperitoneally with 5 mg/kg AC prior to IR; (4) IR + AC10 group: mice were injected intraperitoneally with 10 mg/kg AC prior to IR. Each mouse in the AC treated groups received the AC injection 18 h before surgery to allow delivery and uptake in the liver. The sham and IR groups received saline injections only.

### Surgical Procedures

All mice were anesthetized by intraperitoneal administration of a mixture of ketamine and xylazine. They were placed on a heating pad to maintain body temperature at 37°C. Stopping the blood supply to the left and median lobes of the liver in the IR groups was accomplished with a microvascular clamp, leading to partial (70%) hepatic ischemia. After 50 min of ischemia, reperfusion was initiated by removing the clamp. Animals were sacrificed for analysis 6 h after reperfusion. These conditions were based on published literature and our own studies. For example, we investigated the time course of hepatic enzyme elevation as indicators of injury, and found that there was maximal elevation between 3 and 8 h post-reperfusion (data not shown), consistent with other reports (Guo et al., 2019; Liu et al., 2019). Thus, we decided to analyze the impact of AC on IR injury at 6 h post-reperfusion. All animals underwent the same experimental procedure except that the sham animals did not undergo vessel occlusion.

### Sample Collection

For most experiments blood samples was collected at 6 h after IR unless otherwise indicated. Serum was isolated after centrifugation at 1,500 × *g* for 10 min and stored at 4°C for same day enzyme activity assays or otherwise kept at −20°C. To analyze tissues, one portion of the left lobe of the liver was fixed in 10% neutral-buffered formalin and the rest was homogenized in 10% (w/v) tissue lysis buffer (20 mM HEPES buffer pH 7.5, 150 mM NaCl, 1× protease inhibitor cocktail, 0.2% Igpal). The homogenate was transferred into a new 1.5 ml tube and centrifuged at 10,000 × *g* for 10 min at 4°C. The supernatant was again transferred into a new 1.5 ml tube and stored at −20°C for lipid analyses.

### Analysis of Liver Enzyme Activities, MDA and Cytokine Levels

The activities of ALT, AST and LDH in mouse serum were measured using microplate assay kits according to the manufacturer’s instructions. The concentration of MDA and the inflammatory cytokine MCP-1 also was measured with MDA assay and MCP-1 ELISA kits, respectively, according to the manufacturer’s instruction.

### Quantification of Ceramide, Sphingosine and S1P in Mouse Liver

Lipid extracts were prepared from liver homogenates by the classic Folch method (Folch et al., 1957) using chloroform/methanol (2:1). The lipid extract was then dried under nitrogen gas and re-dissolved in a 2% Igepal solution. The ceramide, sphingosine, and S1P levels were measured as previously described (He et al., 2009, 2017). Briefly, to determine the total ceramide content the 2% Igepal lipid extract was resuspended in a hydrolysis buffer containing 0.2 mg/ml of recombinant AC. This completely hydrolyzed the ceramide in the extract to sphingosine. The produced sphingosine was then derivatized into a fluorescent product after being reacted with a fluorogenic compound, NDA, and analyzed using an Acquity H-Class UPLC system (Waters) monitored at excitation and emission wavelengths of 252 and 483 nm, respectively. Quantification of the fluorescent sphingosine was calculated using the Waters Empower software according to a standard curve derived from commercial sphingosine. For quantification of sphingosine, the same procedure was used except that the ceramide hydrolysis step was excluded (i.e., no recombinant AC was added). In this way endogenous sphingosine present in the lipid extract could be derivatized directly with NDA and quantified by UPLC. For S1P quantification the same procedure was used since the derivatized sphingosine and S1P peaks could be readily separated and individually quantified by UPLC.

### Analysis of SMase Activities in Mouse Serum and Liver

The acid and neutral sphingomyelinases (ASM and NSM) activities were measured in serum and liver homogenates as previously described (He et al., 2003a). Briefly equal volumes of serum or liver homogenates were mixed with assay buffers for ASM (0.2 mM Bodipy-FL C12 sphingomyelin, 0.2 M sodium acetate buffer, pH 5.0, 0.2 mM ZnCl_2_) or NSM (0.2 mM Bodipy-FL C12 sphingomyelin, 50 mM HEPES buffer, pH 7.2, 5 mM MgCl_2_), respectively. After 30 min of incubation at 37°C, the reaction mixtures were analyzed using an Acuity H-Class UPLC system equipped with a BEH-amide 1.7 μm column (2.1 mm × 50 mm) and monitored at excitation and emission wavelengths of 500 and 520 nm, respectively. ASM and NSM activities were determined based on quantitation of the area under the peak of the product, Bodipy-FL C12 ceramide, according to a standard curve.

### Analysis of SPHK Activities in Mouse Liver

SPHK1 and SPHK2 activities were determined as described previously (Plohn et al., 2018) with modification. Briefly, equal volumes of liver homogenates were incubated with SPHK1 assay buffer (50 μM NBD-sphingosine, 100 mM HEPES, pH 7.5, 250 mM NaCl, 2 mM ATP, 30 mM MgCl_2_, 1 mM deoxypyridoxine, 1 × protease and phosphatase inhibitor cocktails, 0.1% TX-100). The SPHK2 assay buffer was the same except that 2 M KCl was added instead of 0.1% TX-100. After 1 h of incubation at 37°C, all reactions were analyzed using an Acuity H-Class UPLC system equipped with a Waters BEH-C18 1.7 μm column (2.1 mm × 30 mm) and monitored at excitation and emission wavelengths of 435 and 525 nm, respectively. The calculations of SPHK1 and SPHK2 activities were based on quantitation of the area under the peak of the product, NBD-sphingosine-1-phosphate, according to a standard curve.

### Histopathology

The left lobes of the liver were collected from individual mice in the sham, IR and treatment groups. One part of the left lobe was fixed in formalin solution and embedded in paraffin. The paraffin-embedded liver tissue was cut into 5 μm sections and stained with hematoxylin and eosin (HE). The stained sections were reviewed under an Olympus BX40 microscope and the pictures were captured by an AmScope digital camera (FMA050) and software. Liver injury was semi-quantified using a modification of a scoring system described previously (Camargo et al., 1997). Briefly, 50 adjacent fields were scored per slice at a 200× magnification to determine the grades, with grade 0 for no evidence of injury, grade 1 for mild injury with nuclear defragmentation, grade 2 for intermediate injury with nuclear pyknosis, and grade 3 for severe injury with karyolysis. In addition to the above, we also analyzed the slides according to the Suzuki scoring criteria (Suzuki et al., 1993), which assesses injury based on congestion, vacuolization, and necrosis. Two independent readers reviewed the slides and were blinded to the treatment groups during the analysis.

### Statistical Analysis

All results were expressed as means ± standard deviation (SD). One-Way Anova and Student’s *t*-test with unequal variance were used to analyze the data. In all comparisons, differences between groups were considered statistically significant according to *P* < 0.05. Two sets of comparisons were made. First, to insure that the surgical methods were inducing IR injury, we assessed changes between the sham and IR group. In addition, we compared changes between the two AC treatment groups with the IR group to assess the impact of AC pretreatment. In all figures the *p*-values are indicated if a significant difference was found.

## Results

### ALT, AST, and LDH Activities

**FIGURE 1:**
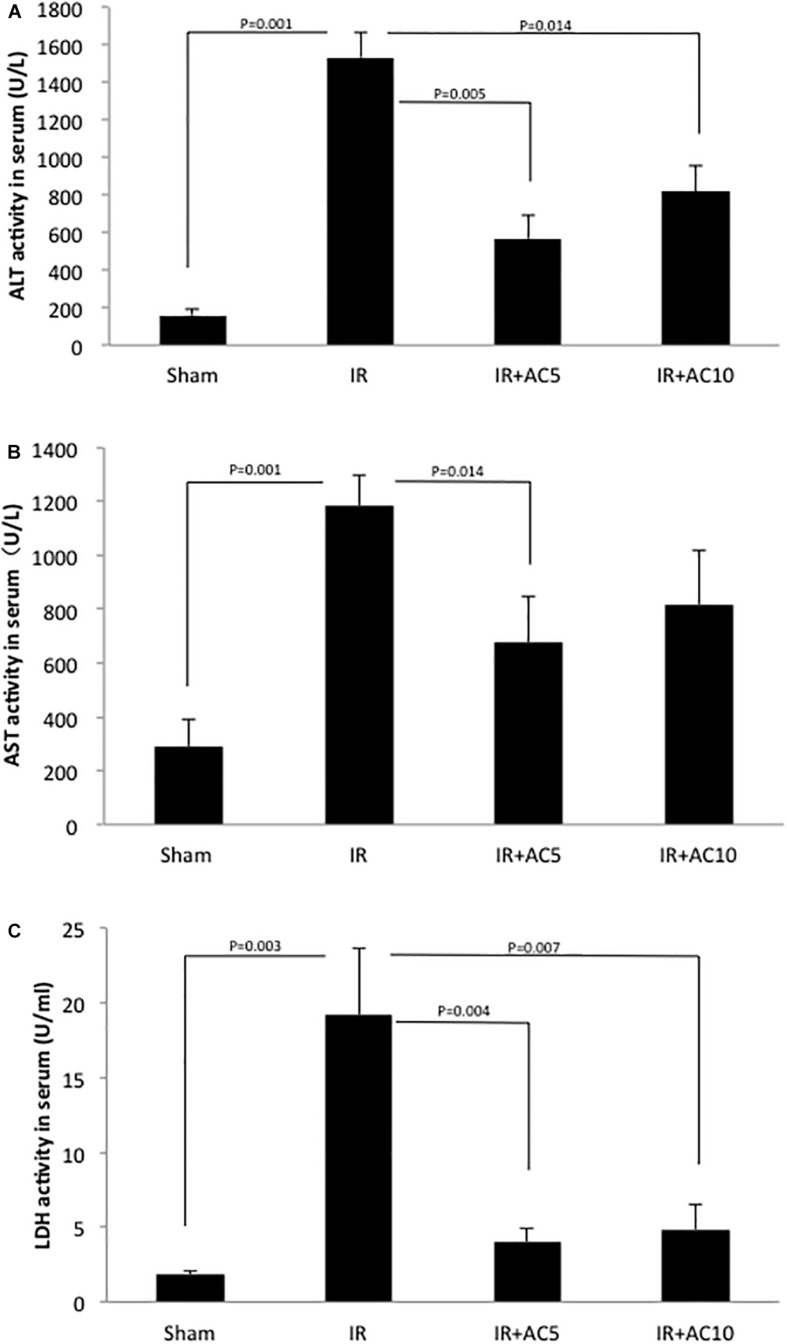
Acid ceramidase (AC) pretreatment prevents the elevation of liver enzymes in serum in response to IR injury. The levels of ALT **(A)**, AST **(B)**, and LDH **(C)** were significantly elevated in the IR group compared to a sham treated group. Single pretreatment with either 5 (AC5) or 10 mg/kg (AC10) of recombinant AC 18 h prior to IR prevented this elevation. Each bar represents the mean value (*n* = 10 mice per group). The standard deviations are shown for each group and *p*-values are indicated for significant comparisons. Measurements were made at 6 h post-reperfusion.

To initially evaluate hepatic IR injury, the activities of several liver enzymes (ALT, AST, and LDH) were determined. After 50 min of ischemia and 6 h of reperfusion, the activities of these three enzymes in serum were all dramatically increased in the IR group compared to the sham group. However, a single pretreatment with AC (5 or 10 mg/kg injected 18 h prior to ischemia), significantly reduced serum ALT, AST, and LDH activities in the IR group (Figures 1A–C). No significant differences were found between the two AC dose groups.

### Ceramide, Sphingosine and S1P Levels

**FIGURE 2:**
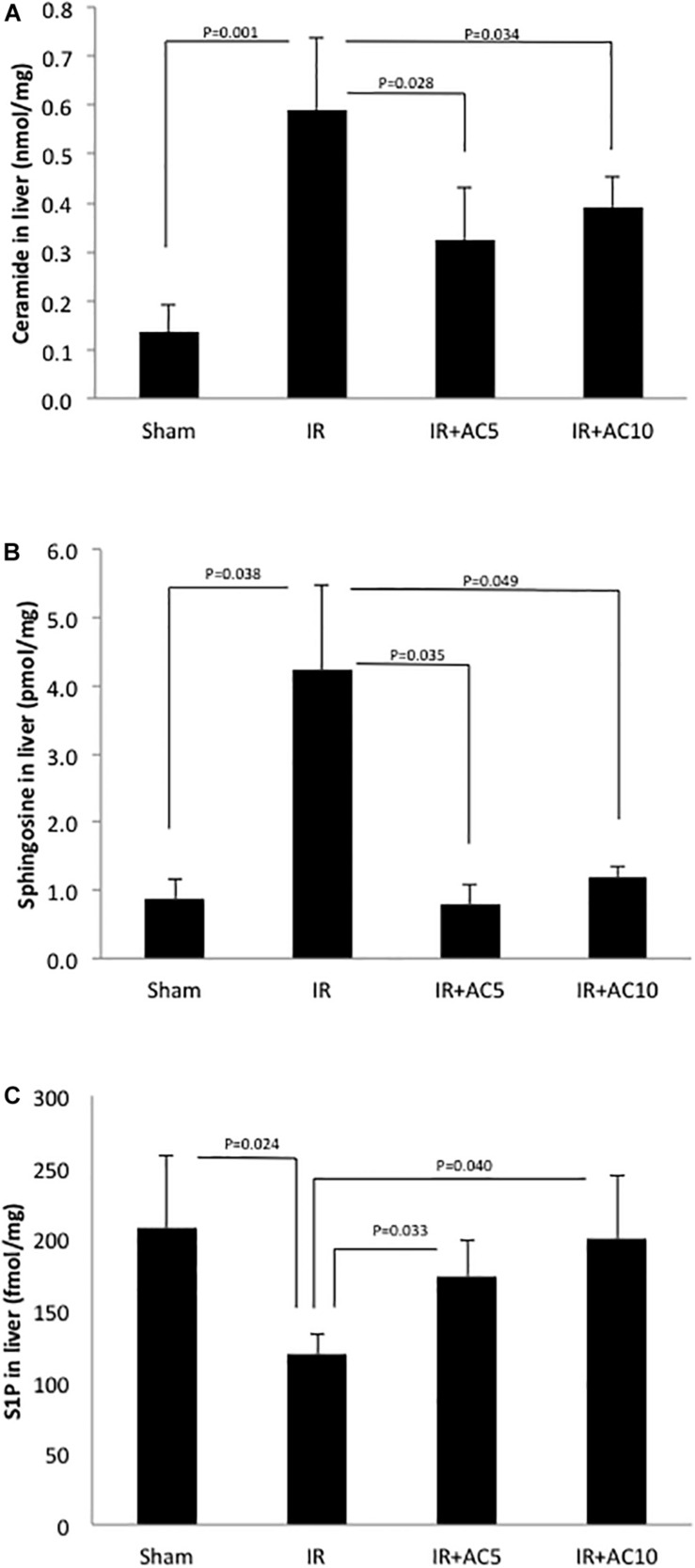
Acid ceramidase pretreatment prevents the elevation of ceramide and sphingosine in the liver response to IR injury, and elevates the levels of S1P. The levels of ceramide and sphingosine were elevated in liver extracts following IR injury compared to the sham group (**A,B**, respectively), while the levels of S1P were reduced **(C)**. Single pretreatment with either 5 (AC5) or 10 mg/kg (AC10) of recombinant AC 18 h prior to IR prevented these changes. Each bar represents the mean value (*n* = 10 mice per group). The standard deviations are shown for each group and *p*-values are indicated for significant comparisons. Measurements were made at 6 h post-reperfusion.

Next, ceramide, sphingosine and S1P levels were analyzed in the liver tissue. After 50 min of ischemia and 6 h of reperfusion, the levels of ceramide were significantly increased compared to the sham group (Figure 2A), similar to that observed with the liver enzymes. Notably, the ceramide levels in the IR groups pretreated with AC (either dose) were significantly lower than those in the untreated IR group. Interestingly, similar results were found for sphingosine (Figure 2B), where IR elevated the levels in liver and pretreatment with AC significantly reduced it. No significant differences in the ceramide and sphingosine levels were found between the two AC dose groups. Since the levels of both ceramide and sphingosine were elevated following IR injury, we then measured S1P, a product of sphingosine and a pro-survival signaling lipid that often counteracts the effects of ceramide. Following IR injury S1P levels in the liver were significantly reduced compared to sham, and pretreatment with AC recovered these levels to near normal (Figure 2C).

### Sphingolipid Metabolizing Enzyme Activities

**FIGURE 3:**
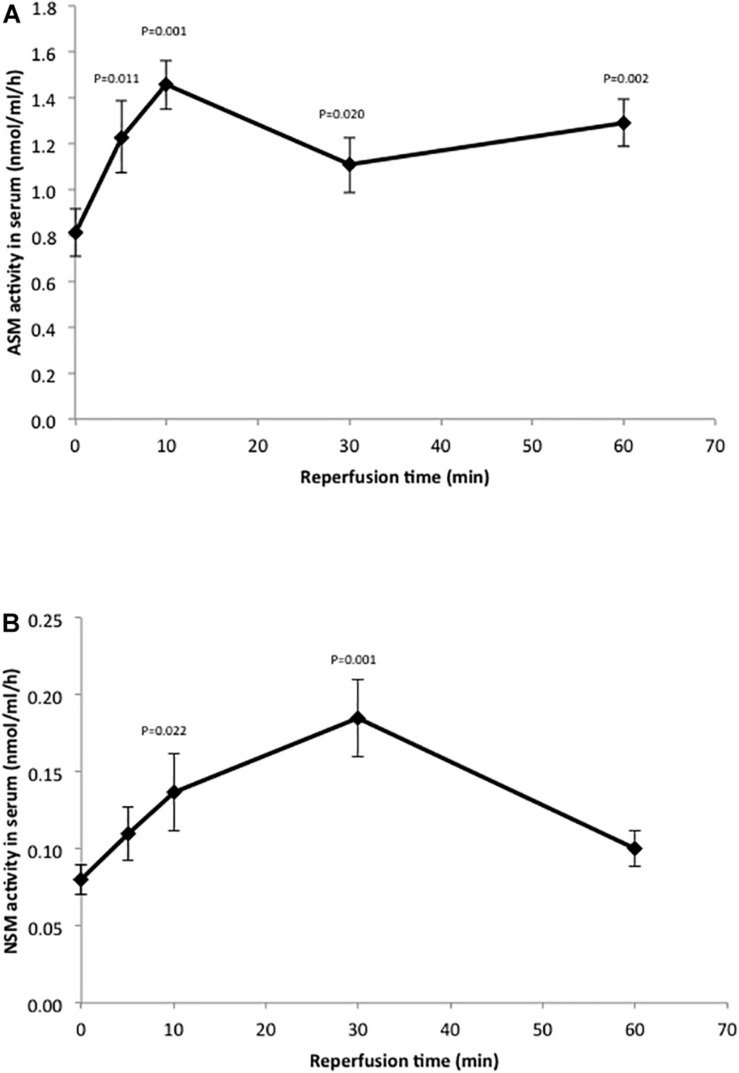
Hepatic IR injury induces rapid changes in ASM and NSM enzyme activities in the serum. ASM **(A)** and NSM **(B)** activities were determined in serum as described in the text. Note the rapid increase in both activities in response to IR injury. The mean activity value at each time point (*n* = 10 mice) is shown, and the standard deviations and *p*-values are indicated as compared to baseline.

The changes in sphingolipid levels we observed in response to IR injury indicated that one or more of the enzymes that metabolize these lipids might be altered as well. Since sphingomyelin hydrolysis is a main source of ceramide in cells, we first measured the activities of two SMases (acid and neutral; ASM and NSM) in the liver under our standard conditions (50 min of ischemia and 6 h of reperfusion). However, we did not see any increases in these activities (Supplementary Figure 1). We then reasoned that since ceramide was elevated at 6 h post-perfusion (Figure 2A), perhaps the change(s) in Smase activities was occurring earlier. Indeed, rapid increases in Smase activities have been shown in response to other stress inducers, leading to the sustained elevation of ceramide (Haimovitz-Friedman et al., 1997). Therefore, we conducted a time course experiment where serum was collected from the animals at 5, 10, 30, and 60 min post-reperfusion. As shown in Figure 3, there was a rapid response to IR injury for both Smases observed within 5 min post-reperfusion. In the case of ASM, the activity maximized at 10 min but remained significantly elevated at 60 min post-reperfusion, while for NSM the activity maximized at 30 min and returned to near baseline by 60 min post-reperfusion.

**FIGURE 4:**
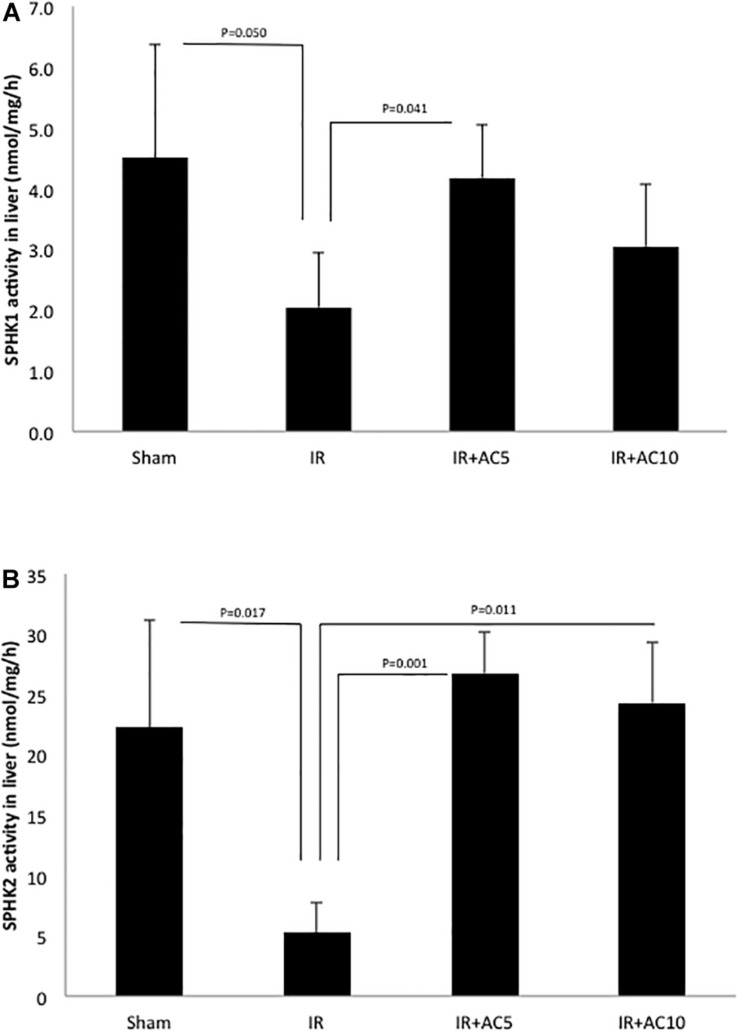
Acid ceramidase pretreatment prevents the reduction of SPHK1 and SPHK2 activities in the liver in response to IR injury. SPHK1 **(A)** and SPHK2 **(B)** activities were determined in liver extracts as described in the text. Note that both activities were significantly reduced following IR injury, and that single pretreatment with either 5 (AC5) or 10 mg/kg (AC10) of recombinant AC 18 h prior to IR prevented these reductions. These findings are consistent with the S1P and sphingosine findings depicted in Figure 2. Each bar represents the mean value (*n* = 10 mice per group). The standard deviations are shown for each group and *p*-values are indicated for significant comparisons. Measurements were made at 6 h post-reperfusion.

We also measured the activities of sphingosine kinases 1 and 2 (SPHK1 and SPHK2), the enzymes responsible for producing S1P from sphingosine. Following 50 min of ischemia and 6 h of reperfusion, we found that the liver activity of both enzymes were significantly reduced compared to sham, and that these activities were recovered after AC pretreatment (Figure 4). Overall, these results were consistent with the observations on ceramide, sphingosine and S1P shown in Figure 2.

### MDA and MCP-1 Levels

**FIGURE 5:**
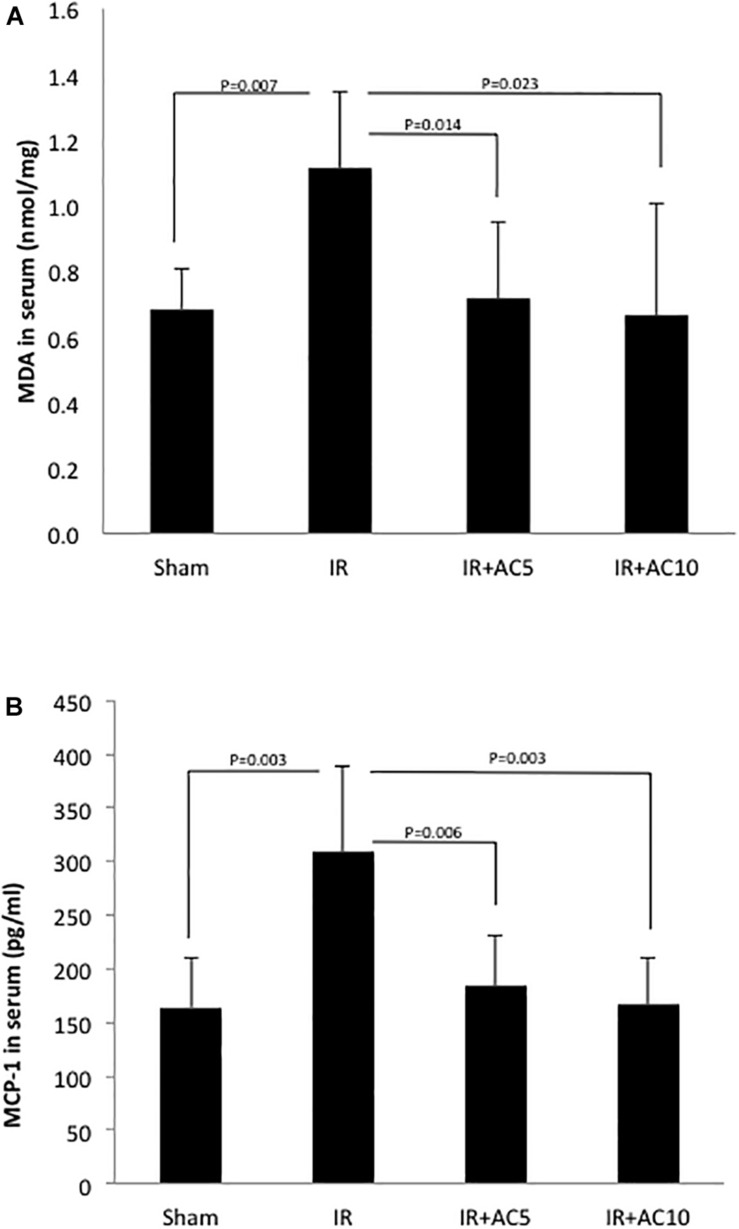
Acid ceramidase pretreatment prevents the elevation of MDA and MCP-1 levels in the serum in response to IR injury. MDA **(A)** and MCP-1 **(B)** were measured in the serum as indicators of oxidative stress and inflammation, respectively. As expected, both increased in response to IR injury. Single pretreatment with either 5 (AC5) or 10 mg/kg (AC10) of recombinant AC 18 h prior to IR prevented these elevations. Each bar represents the mean value (*n* = 10 mice per group). The standard deviations are shown for each group and *p*- values are indicated for significant comparisons. Measurements were made at 6 h post-reperfusion.

Next, we assessed a marker of oxidative stress (MDA) in the serum from these animals, and found a significant elevation in response to IR injury and correction to normal in the groups pretreated with AC (Figure 5A). To assess the effects on inflammation, we determined the levels of the chemokine MCP-1 in the serum, and found that as with MDA after 50 min of ischemia and 6 h of reperfusion the serum MCP-1 concentration in the IR group was dramatically increased compared to the sham, and that AC pretreatment markedly reduced these levels (Figure 5B). No differences in MDA or MCP-1 levels were observed between the two AC dose groups.

### Morphological Changes

**FIGURE 6:**
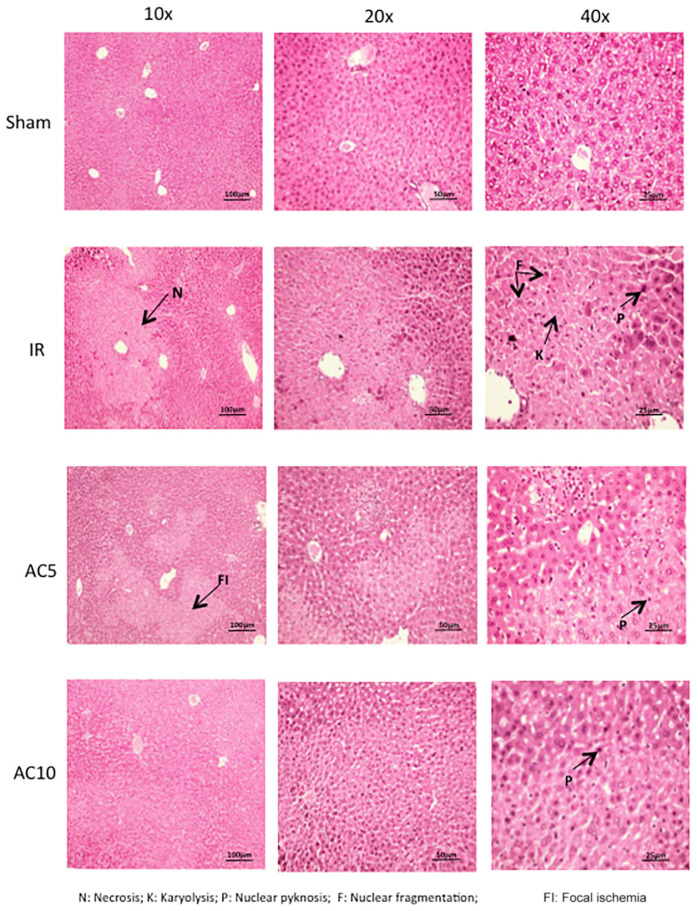
Histologic changes in the liver following IR injury and in response to AC pretreatment. Representative images are shown for each group. Arrows indicate areas of nuclear defragmentation, nuclear pyknosis, and karyolysis. Note that pretreatment with AC prevented many of these changes. Images were selected from over 50 sections analyzed from each mouse (*n* = 10 per group). Sections were prepared at 6 h post-reperfusion.

**FIGURE 7:**
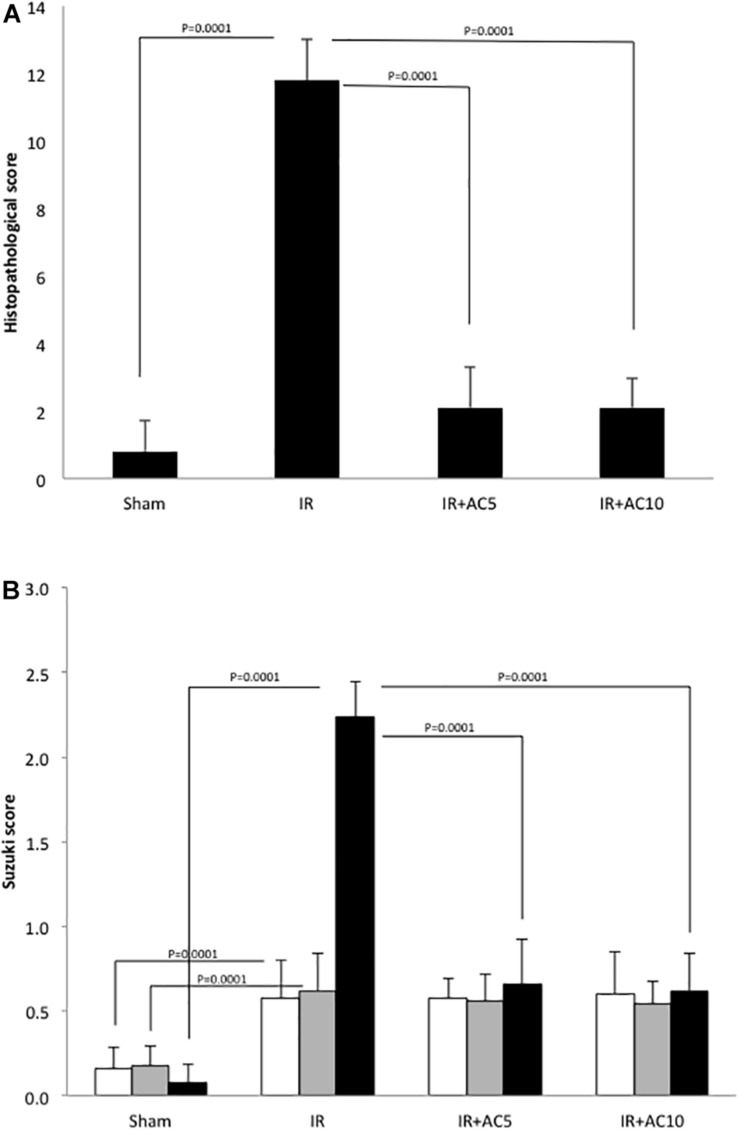
Semi-quantitative scoring of the histologic changes in the liver following IR injury and in response to AC pretreatment. **(A)** 50 adjacent fields per mouse (*n* = 10 mice per group) were scored per slice at a 200× magnification to determine the grades, with grade 0 for no evidence of injury, grade 1 for mild injury with nuclear defragmentation, grade 2 for intermediate injury with nuclear pyknosis, and grade 3 for severe injury with karyolysis. The readers were blinded to the treatment groups during the analysis. Each bar represents the mean value; *p*-values comparing the groups and the standard deviations are indicated. **(B)** The same slides were assessed according to the scoring system of Suzuki et al. (1993). The readers also were blinded to the treatment groups during the analysis. White bars indicate congestion; gray bars indicate vacuolization, and black bars indicate necrosis. Standard deviations and *p*-values also are shown.

Lastly, hepatic pathological changes were evaluated from HE stained liver sections. Compared with the sham group, after 50 min of ischemia and 6 h of reperfusion the liver tissue in the IR group displayed obvious areas of hepatocellular necrosis around the central vein characterized by the presence of nuclear fragmentation, pyknosis, apoptotic bodies and karyolysis (Figure 6). AC pretreatment attenuated the IR-induced liver damage, as demonstrated by less nuclear pyknosis and karyolysis. Semi-quantitative assessment of hepatic histologic injuries showed significantly lower scores in the AC-treated IR groups compared to the IR group alone (Figure 7A), consistent with the liver enzyme findings shown in Figure 1. In addition, the liver sections were analyzed according to the scoring system of Suzuki that assesses three main injury outcomes, congestion, vacuolization and necrosis (Suzuki et al., 1993). High levels of necrosis were evident in the livers of mice subjected to IR, consistent with previous findings (Yaron et al., 2019), and this was significantly reduced in the IR animals receiving AC pretreatment (Figure 7B). In all analyses the readers were blinded to the treatment groups.

## Discussion

Hepatic IR injury occurs in a wide range of clinical settings. For example, when the blood supply is interrupted to the liver during surgical procedures, the cells and tissue are deprived of oxygen and nutrients, leading to hepatocyte cell damage and tissue breakdown. Paradoxically, after blood flow is restored to the ischemic area, rather than repairing the tissue more severe damage often occurs. Extensive investigations have demonstrated that such hepatic IR injury results from activation of multiple oxidative stress and inflammatory pathways, leading to hepatic cell apoptosis and necrosis.

The mechanisms underlying IR injury are complex and involve the interaction of various molecular pathways. Therefore, to mitigate the ensuing tissue damage a wide range of therapeutic strategies have been investigated, including ischemic pre/post-conditioning during surgery, the use of pharmacological agents such as anti-inflammatory drugs and antioxidants, and other treatments including RNA interference, microRNA, stem cell transplantation, etc. However, no ideal approach exists and IR injury remains a major issue in surgical and other clinical settings.

A growing body of evidence has demonstrated that the activation of Kupffer cells and neutrophils, release of pro-inflammatory cytokines, and production of ROS leads to ceramide accumulation in the liver during hepatic IR injury. Moreover, recent data has shown that the administration of SMase inhibitors decreased ceramide accumulation during hepatic IR and attenuated cell necrosis and tissue damage (Fan et al., 2016; Tuzcu et al., 2017), as did inhibition of the *de novo* ceramide synthesis pathway (Cuzzocrea et al., 2008; Reforgiato et al., 2016; Bonezzi et al., 2019). In addition, the generation of S1P, which is known to counteract the effects of ceramide, prevented IR injury as well (Li et al., 2020). Collectively, these results suggest that ceramide plays an important role in IR-induced liver damage, and that the modulation of ceramide levels may be a potential therapeutic target.

Acid ceramidase is a ceramide hydrolase first identified in 1963 (Gatt, 1963). An inherited deficiency of this enzymatic activity leads to two rare clinical conditions in man: Farber disease (Sugita et al., 1972) and Spinal Muscular Atrophy with Myoclonic Epilepsy (Jankovic and Rivera, 1978). The first purification of AC was from human urine in 1995 (Bernardo et al., 1995), and the first production of recombinant AC was in Chinese Hamster ovary cells in 2003 (He et al., 2003b). Recombinant AC is currently being developed for the treatment of Farber disease, and can be readily internalized by cells and reduce ceramide levels (He et al., 2003b, 2017). Importantly, pharmacologic studies in animal models have shown that the liver is the major site of enzyme uptake following systemic administration, suggesting its utility in the management of liver IR injury. As with other lysosomal enzymes, uptake of the exogenous enzyme into cells is via mannose-6-phosphate receptors that interact with terminal mannose-6-phosphate residues present on the enzyme’s N-linked oligosaccharide side chains. Optimal uptake of recombinant AC into the liver occurs 12–24 h after injection (He et al., 2017), leading us to evaluate its impact on IR injury at 18 post-injection.

In addition to Farber disease, the therapeutic potential of recombinant AC also has been investigated in other diseases where ceramide pathology has been demonstrated, for example to reduce inflammation/infection in Cystic Fibrosis and to assist in cartilage repair after joint injury (Pewzner-Jung et al., 2014; Xia et al., 2015; Frohbergh et al., 2016; He et al., 2017; Becker et al., 2018). In the current work we have focused on investigating the potential protective effects of recombinant AC on hepatic IR injury, and further understanding the underlying mechanism(s) contributing to these effects.

The enzymes ALT, AST, and LDH are common indicators of hepatocyte injury, and have important roles in hepatic pathophysiology. As expected, we found markedly elevated serum levels of these enzymes in IR-induced mice compared to those in a sham group (Figure 1). Notably, the levels of all three enzymes were significant lower in IR groups receiving a single pretreatment with recombinant AC, indicating that AC pretreatment played a protective role in hepatic cell injury during IR. These results were in accord with the histopathological changes shown in Figures 6, 7.

During hepatic IR injury the generation and release of inflammatory factors and ROS are among the earliest and most consistent pathologic changes; these, in turn, activate SMases and/or *de novo* synthesis pathways to produce ceramide (Alessenko et al., 2002; Llacuna et al., 2006; Aslan et al., 2014; Fan et al., 2016). The activation of SMases in response to stress is generally very rapid, within minutes of exposure, while the resulting changes in ceramide and downstream lipids can be much more long-lasting (Haimovitz-Friedman et al., 1997). In this study the activities of acid and neutral SMases were carefully monitored in response to hepatic IR injury. Consistent with previous observations, at 6 h post-reperfusion we did not observe significant increases in either Smase activity in the livers of IR mice compared to sham (Supplementary Figure 1), despite the fact that the ceramide levels were highly elevated. We then performed a time course analysis in the serum of IR treated mice, and found that the acid SMase activity exhibited a rapid response to IR (within 5 min post-reperfusion) that lasted for up to 1 h. This was also in agreement with previous findings (Llacuna et al., 2006). Similarly, neutral SMase activity rapidly increased post-reperfusion, although by 1 h this activity had returned to baseline.

Together, these findings indicate that rapid elevation of Smase activities contributes to the ceramide production observed following IR. Importantly, the results do not preclude the possibility that other pathways (e.g., *de novo* synthesis) also play a role in the ceramide response, a hypothesis that could be investigated in future studies. It will also be of interest in the future to look for other systemic changes, including changes in serum sphingolipid levels and/or sphingolipid-metabolizing enzymes. In the current study we focused on sphingolipid changes in the liver since our primary goal was to evaluate the impact of AC pretreatment on liver IR injury, and the majority (up to 90%) of the systemically injected AC is delivered to the liver within 12–24 h where it will degrade ceramide (He et al., 2017).

MCP-1 is a potent monocyte-attracting chemokine, and partly responsible for the recruitment of blood monocytes into sites of inflammation (Deshmane et al., 2009). Previous studies documented the interaction of MCP-1 and SMases in other disease settings, including atherosclerosis and infectious diseases (von Bismarck et al., 2012; Lallemand et al., 2018). Therefore, we determined the level of MCP-1 released into serum during hepatic IR, and whether AC pretreatment mitigated this response. In line with the SMase activities, the concentration of MCP-1 increased by 30 min post-reperfusion (data not shown), and was dramatically higher by 6 h (Figure 5B). Of note, AC pretreatment prevented the IR-induced increase in MCP-1 levels.

Due the critical role of ceramide in cell metabolism and survival, levels of this lipid are carefully regulated. This occurs at both the genetic and post-translational level. For example, in addition to Smases and *de novo* synthetic enzymes, which produce ceramide, endogenous ceramidases, including AC, hydrolyze this lipid to sphingosine, which is further phosphorylated by one of two sphingosine kinases to form S1P. S1P is a protective signaling lipid that counters many of the toxic effects of ceramide.

Here we found that ceramide was dramatically elevated in the liver of IR treated mice compared to the sham group, in agreement with many previous reports implicating this lipid in IR injury (Figure 2). This is likely due, at least in part, to the rapid elevation of SMases (Figure 3). Surprisingly, the level of sphingosine in the liver of the IR group also was significantly higher than those in sham group (Figure 2B), indicating that the downstream metabolism of this lipid may be disrupted as well. Indeed, we found that the activity of SPHK1 and 2 were both significantly reduced in the liver following IR injury (Figure 4), likely contributing to the elevation of sphingosine and a reduction in the protective lipid S1P (Figure 2C). Pretreatment of mice with recombinant AC prior to IR corrected all of these sphingolipid abnormalities in the liver by reducing ceramide and sphingosine levels, and elevating S1P via activation of the sphingosine kinases. In these studies we could not monitor the levels of endogenous ceramidases since we were injecting mice with high levels of AC, which would confound the results.

The role of S1P in liver injury has been extensively investigated (Chen and Hu, 2020). In general, this signaling lipid plays a protective role to injury by countering the toxic effects of ceramide, including apoptosis and necrosis. However, S1P also activates inflammatory pathways, which in some cases could contribute to or even accelerate injury if not properly regulated. Multiple studies have shown that the activities of SPHK1 and 2 were significantly reduced during IR, and/or that activation of these enzymes lead to the production of S1P and protection of the liver against injury (Nojima et al., 2016; Du et al., 2017; Kang et al., 2019; Li et al., 2020). SPHK1 mutation also sensitized mice to cardiac IR injury (Jin et al., 2007).

In contrast, two reports demonstrated that SPHK1 knockout (Qiang et al., 2019) or SPHK2 inhibition (Shi et al., 2012) protected mice from IR injury. Future studies should be performed to fully clarify the role of these two enzymes and S1P in IR, but in our studies we show that IR injury leads to a reduction of both SPHK1 and SPHK2 activities in the liver, leading to elevation of sphingosine and reduction of S1P. Sphingosine, like ceramide, may be toxic to the liver (Kwong et al., 2019), and overall it is likely that the injury we observe with IR is due to a combination of ceramide and sphingosine elevation, and potentially reduced S1P levels as well.

Importantly, in this manuscript we show for the first time that pretreatment with AC, which degrades the accumulated ceramide in the liver after IR, also corrects these downstream changes by normalizing sphingosine and S1P, contributing to prevention of the IR-induced liver injury. The effects on sphingosine and S1P are likely due, at least in part, to the elevation of SPHK activities. As noted above, we focused this study on lipid and enzyme changes in the liver, but in the future it will be interesting to examine systemic changes as well, including the release of S1P into the blood. We also could apply mass spectrometry-based lipidomics analysis to gain a broader picture of the lipid changes induced by IR and corrected by AC pretreatment.

Acid ceramidase administration has considerable advantages over other approaches to mitigate ceramide generation during hepatic IR injury. Most notably, not only does AC prevent ceramide accumulation, but it also corrects the downstream sphingolipid changes that occur in response to IR injury, including the elevation of sphingosine and reduction of S1P. In contrast, while SMase inhibitors may slow the production of ceramide, their effects on the downstream lipid changes are unknown. Ceramide is also generated in cells by multiple mechanisms (e.g., *de novo* synthesis), and SMase inhibitors can only prevent one, the degradation of sphingomyelin into ceramide. AC, on the other hand, will degrade ceramide generated by multiple mechanisms. In the future it will be interesting to examine the *de novo* pathway in response to liver IR injury, as well as the impact of AC pretreatment on this pathway. In addition, SMase inhibitors have off target effects due their lack of specificity, and their uptake by the liver is variable. AC is a highly specific ceramide hydrolase that is rapidly taken up by the liver by well-defined mechanisms. Indeed, over 90% of the enzyme administered by IV injection can be found in the liver within 24 h (He et al., 2017).

**FIGURE 8:**
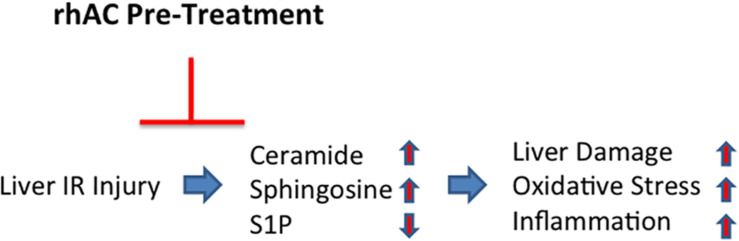
Schematic depiction showing the effects of AC pretreatment on liver IR injury. A single injection of recombinant AC 18 h prior to IR injury prevented the IR-induced changes in several sphingolipids (i.e., elevation of ceramide and sphingosine; reduction of S1P), leading to the prevention of oxidative stress, inflammation and tissue damage.

In summary, the findings presented herein indicate that AC plays an important role in protecting the liver against IR-induced cell injury, leading to inhibition of systemic inflammatory responses, reduction in oxidative stress, and reduction in hepatic injury. Our study further identifies ceramide as a major therapeutic target in IR injury, and suggests that the recombinant AC might be a novel therapeutic approach to prevent hepatic IR injury, particularly since it is very efficiently delivered to the liver and also corrects the sphingosine and S1P abnormalities induced by IR (Figure 8). Recombinant AC is currently being manufactured for clinical use in Farber disease patients, which could facilitate its rapid repurposing for the prevention of hepatic IR injury.

The studies presented here are proof-of-concept for this novel approach. In principle, it could be easily applied to patients by administering a single injection of recombinant AC 18–24 h prior to liver surgery and/or induction of hepatic IR. Future studies should more carefully assess the dose response of these effects, as well as more broadly assess the lipid changes induced by AC pretreatment. Since we did not see a difference in the response between the 5 and 10 mg/kg dose groups, this may indicate that we have saturated the effect and could titrate the dose down further to obtain a minimal effective dose.

## Disclosures

ES and XH are inventors on a patent filed by the Icahn School of Medicine describing the use of recombinant AC for prevention of IR injury.

## Data Availability Statement

The raw data supporting the conclusions of this article will be made available by the authors, without undue reservation, to any qualified researcher.

## Ethics Statement

The animal study was reviewed and approved by all experiments in this study were performed at the Icahn School of Medicine under a protocol (#98-0089) approved by the Institutional Animal Care and Use Committee.

## Author Contributions

YJ and XH contributed equally to this work and participated in the design and execution of all experiments. CS contributed to the execution of experiments and interpretation of results. ES oversaw all experiments, interpretation of the results, and writing of the manuscript. YJ, XH, BY, and CS reviewed the manuscript prior to submission and made modifications as required. All authors contributed to the article and approved the submitted version.

## Conflict of Interest

The authors declare that the research was conducted in the absence of any commercial or financial relationships that could be construed as a potential conflict of interest.

## Supplementary Material

Supplementary Figure 1 Acid ceramidase pretreatment has no effect on ASM and NSM enzyme activities in the liver at 6 h post IR injury. Single pretreatment with either 5 (AC5) or 10 mg/kg (AC10) of recombinant AC 18 h prior to IR had no effect on liver ASM **(A)** and NSM **(B)** activities when measured 6 h post-reperfusion. This led us to perform an early time course analysis for these activities in the serum, finding early elevation of both activities in response to IR injury (Figure 3). This is consistent the elevation of ceramide observed in Figure 2. Each bar represents the mean activity value (*n* = 10 mice per group). Standard deviations for each group are shown, as is the *p*-value for the only comparision that was significant (reduction in ASM activity comparing the sham and IR groups).
