# Supplementary figures and images for: Acid Ceramidase Protects Against Hepatic Ischemia/Reperfusion Injury by Modulating Sphingolipid Metabolism and Reducing Inflammation and Oxidative Stress
Source: Front Cell Dev Biol. 2021 May 6;9:633657. doi: 10.3389/fcell.2021.633657 (PMC8134688; doi:10.3389/fcell.2021.633657)

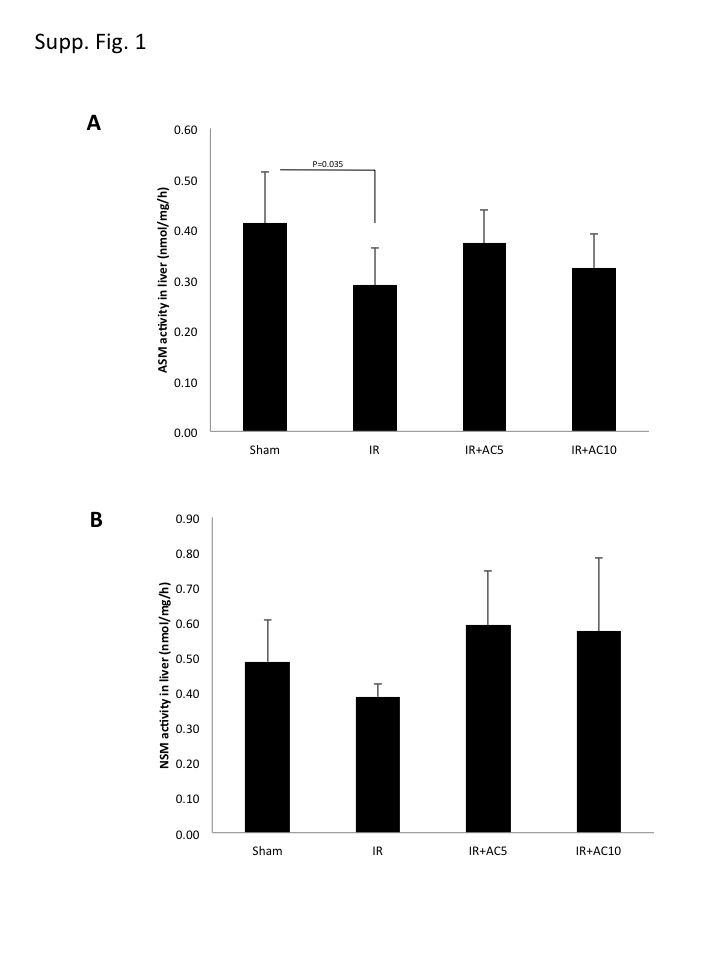

Supplement: Supplementary Figure 1 — Acid ceramidase pretreatment has no effect on ASM and NSM enzyme activities in the liver at 6 h post IR injury. Single pretreatment with either 5 (AC5) or 10 mg/kg (AC10) of recombinant AC 18 h prior to IR had no effect on liver ASM (A) and NSM (B) activities when measured 6 h post-reperfusion. This led us to perform an early time course analysis for these activities in the serum, finding early elevation of both activities in response to IR injury (Figure 3). This is consistent the elevation of ceramide observed in Figure 2. Each bar represents the mean activity value (n = 10 mice per group). Standard deviations for each group are shown, as is the p-value for the only comparision that was significant (reduction in ASM activity comparing the sham and IR groups). [file Image_1.tiff]
